# Phenotypic plasticity in the pancreas: new triggers, new players

**DOI:** 10.1016/j.ceb.2017.11.014

**Published:** 2017-12

**Authors:** Theoni Ingrid Demcollari, Ana-Maria Cujba, Rocio Sancho

**Affiliations:** Centre for Stem Cells and Regenerative Medicine, Faculty of Life Sciences & Medicine, King's College London, Guy's Hospital, 28th Floor, Tower Wing, London SE1 9RT, UK

## Abstract

The pancreas has a very limited regenerative potential during homeostasis. Despite its quiescent nature, recent *in vivo* models suggest a certain degree of regeneration and cellular interconversion is possible within the adult pancreas. It has now become evident that cellular plasticity can be observed in essentially all cell types within the pancreas when provided with the right stress stimuli.

In this review, we will focus on the latest findings uncovering phenotypic plasticity of different cell types in the pancreas, the molecular mechanisms behind such plasticity and how plasticity associated with pancreatic or non-pancreatic cells could be harnessed in the generation of new insulin-producing beta cells.

## Highlights

• Cellular interconversions are observed in the adult murine pancreas upon different stimuli.
• The different cell types of the pancreas are heterogeneous.
• Ngn3 activation is observed in most of the cellular interconversions within the pancreas.
• Inter-organ plasticity can be achieved when overexpressing Ngn3, Pdx1 and MafA.
• *Ex vivo* models provide a novel platform to explore ways of generating new beta cells.

**Current Opinion in Cell Biology** 2017, **49**:38–46

This review comes from a themed issue on **Cell differentiation and disease**

Edited by **Magdalena Gotz** and **Senthil Muthuswamy**

For a complete overview see the Issue and the Editorial

Available online 8th December 2017

**https://doi.org/10.1016/j.ceb.2017.11.014**

0955-0674/© 2017 Elsevier Ltd. All rights reserved.

## Introduction

The precise control of tissue homeostasis is essential for multicellular organisms. Tissue homeostasis maintenance has been classically attributed to proliferation of terminally differentiated cells and to differentiation of dedicated adult stem cells. However, it has now become clear that cell plasticity is an additional player in tissue homeostasis, especially after injury [1]. Cell plasticity — that is, the ability of one cell type to convert into another by lineage reversion (dedifferentiation) or direct differentiation (transdifferentiation) — has been extensively observed in highly dynamic tissues such as skin and intestine [2]. Conversely, observing cellular plasticity events in less active tissues, such as the pancreas, has been more challenging.

**Figure 1:**
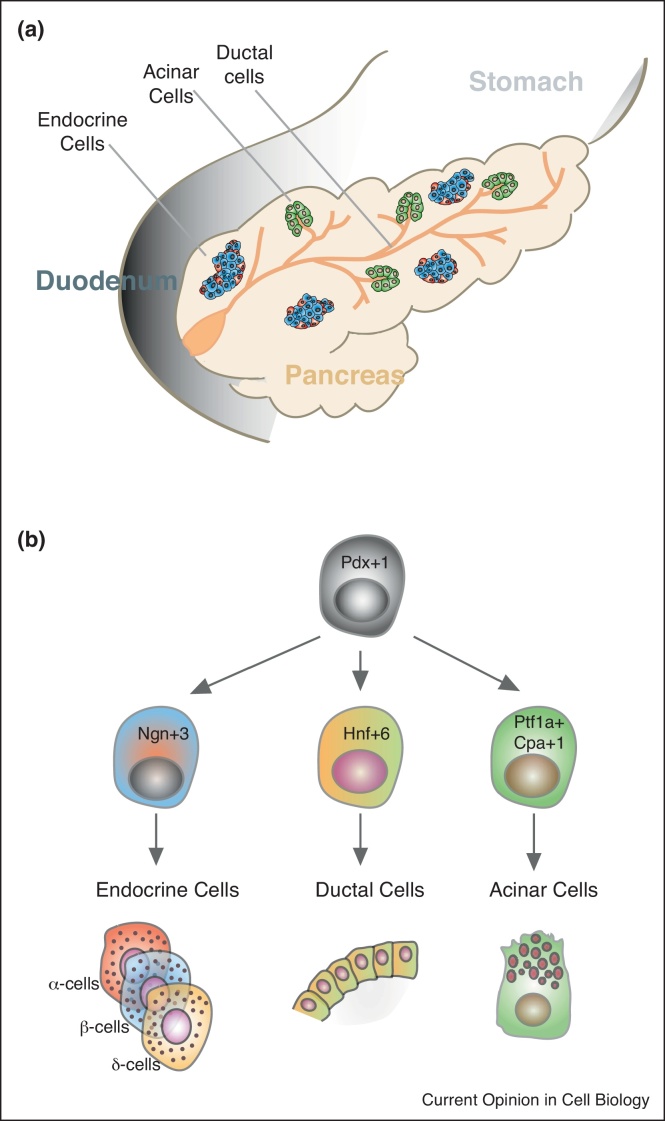
Pancreas scheme. **(a)***Schematic overview of the pancreatic compartments, consisting of exocrine and endocrine parts.* The acinar and ductal cells compose the exocrine pancreas; the acinar cells secrete digestive enzymes that are channeled to the small intestine via the pancreatic ductal tree. The endocrine cells, confined to the islets of Langerhans, secrete glucose-regulating hormones into the bloodstream. **(b)***Development of the three terminally differentiated cell types found in the adult pancreas*. Endocrine, ductal and acinar cells arise from the Pdx1+ embryonic progenitors during development. Transcription factors such as Ngn3 (endocrine cells), Ptf1/Cpa1 (acinar cells) and Hnf6 (ductal cells) are key in coordinating cell fate decisions during embryogeneis.

In sharp contrast to the dynamism of epidermal and intestinal cells, pancreatic cells do not regenerate continuously. The pancreas is a mixed gland composed of exocrine (ductal and acinar cell) and endocrine (alpha, beta, pp, delta and epsilon cell) parts. Exocrine cells fulfil digestive functions. Acinar cells specialise in producing and releasing enzymes that are guided to the duodenum through a network formed by ductal cells. Endocrine cells, physically confined to the islets of Langerhans, regulate glucose metabolism by secreting different hormones to the bloodstream. Insulin (from beta cells), glucagon (from alpha cells) and somatostatin (from delta cells) are essential hormones produced in the pancreatic islets (Figure 1a) [3]. Loss of beta cells in type-1 diabetes is an irreversible process due to the quiescent nature of the pancreas during homeostasis. Therefore, exploiting new sources to generate beta cells has become the main therapeutic strategy in regenerative medicine for diabetes.

The three terminally differentiated and fully specialised cell types in the adult pancreas (acinar, ductal and endocrine cells) arise from Pdx1+ embryonic progenitors during development [4]. These cells proliferate and differentiate constantly during pancreatic embryogenesis, with cell fate decisions meticulously regulated by transcription factors such as Neurogenin 3 (Ngn3) (endocrine cells), pancreas transcription factor 1 complex (Ptf1)/Carboxypeptidase A1 (Cpa1) (acinar cells) and Hepatic Nuclear Factor 6 (Hnf6) (ductal cells) (Figure 1b) [4, 5, 6]. While adult differentiated pancreatic cells are relatively quiescent in basal homeostasis, certain conditions activate a regenerative program based on expansion of existing cells. Pregnancy triggers proliferation of beta cells, and inflammation and oncogenic stress induce acinar cell proliferation [7, 8, 9].

This traditional view of the pancreas in which terminally differentiated cells can give rise only to cells of the same type has been challenged as a result of multiple experimental advances, particularly in single cell RNA-seq analysis [10••, 11•], the use of more robust and specific lineage tracing models [4], improved detection methods and a greater variety of stress stimuli [12]. These latest findings have revealed that different cell types in the pancreas are heterogeneous, and they harbour different plasticity potential. Furthermore, the extent of plasticity for each specific cell type mostly depends on the trigger used. This is best exemplified by acinar cells, which if challenged with inflammation or oncogenic stress will proliferate and transdifferentiate to ductal-like progenitors, but which will adopt a functional beta cell fate if depletion of existing beta cells is combined with transient cytokine administration [13, 14].

Here, we will focus on recently uncovered regenerative processes that are involved in phenotypic plasticity of pancreatic cells both *in vivo* and *in vitro*, with special emphasis on plasticity towards a beta cell fate.

## *In vivo* pancreas plasticity

### Intra-islet plasticity

**Figure 2:**
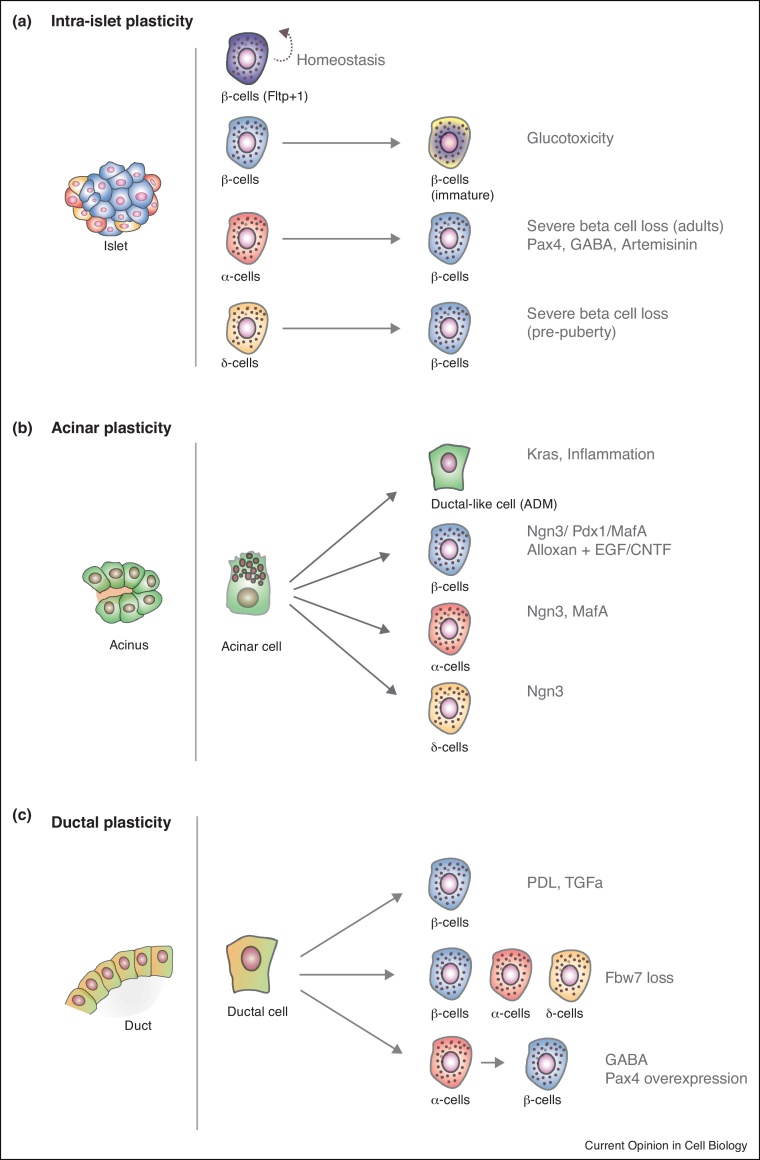
Pancreas plasticity *in vivo*: intra-islet, acinar and ductal plasticity. **(a)***Intra-islet cell plasticity.* Experimental and pathologic conditions can lead to interconversion between islet cell types. Specifically, various studies have shown that Fltp1 expression partly drives heterogeneity within beta cells, overexpression of Pax4 induces alpha-to-beta-cell conversion and delta cells spontaneously transdifferentiate into beta cells following beta cell ablation. **(b)***Acinar cell plasticity.* Inflammation and oncogenic stress can cause transdifferentiation of acinar cells towards ductal-like cells with progenitor abilities (acinar-to-ductal metaplasia). Furthermore, acinar to beta cell plasticity has been artificially induced by various strategies such as the adenoviral infection of acinar cells with the proendocrine factors Ngn3/Pdx1/MafA, *in vivo*. Acinar to alpha and delta cell fate conversion could be controlled by Ngn3 and MafA expression patterns. **(c)***Ductal cell plasticity.* Pancreatic duct ligation was the first trigger to demonstracte the ductal to beta conversion. Diphtheria toxin-induced depletion of acinar and beta cells can drive beta cell mass regeneration from the surviving ductal cells. Also, TGFa overexpression and pancreatic ductal deletion of Fbw7 were shown to convert ductal cells to beta cells. Moreover, activation of Stat3 and Ngn3 in ductal cells induces endocrine lineage transdifferentiation. Pax4 overexpression in alpha cells has also shown ductal-to-beta cell plasticity.

Pregnancy was one of the first stimuli described to affect beta cell numbers, and it is thought to induce equal expansion of the beta cell population [7]. Recent data have demonstrated a clear heterogeneity within beta cells, distinguished by Flattop (Fltp1) expression, which partly drives their plastic behaviour (Figure 2a). Tracing experiments using Fltp1-venus reporter transgenic mouse demonstrated that Fltp1 subdivides endocrine cells into two populations and distinguishes proliferation-competent from mature beta cells [15^••^]. In addition to proliferation, dedifferentiation of beta cells to immature Ngn3-expressing beta cells happens under glucotoxic conditions and this process is reverted when glucose levels are restored [16]. This is consistent with the recent notion derived from single cell RNA-seq analysis of different subtypes of beta cells coexisting in the islets [10••, 11•]. Different RNA-seq subtypes could represent cells with different plasticity potentials, an idea that should be formally tested in the near future.

Intercellular conversions within the islets are observed when severe diabetes is induced in rodents (Figure 2a). Complete ablation of beta cells combined with exogenously maintained normoglycemia in mice results in alpha cells transdifferentiating to beta cells without proliferation [17]. This effect is observed from puberty through to adulthood. The generated alpha-derived beta cells are fully functional. However, alpha cells are unable to recover the complete loss of beta cells before puberty, but delta cells are competent to transdifferentiate to beta cells [18]. This data suggests the existence of different temporal windows permissive to alpha and delta cell plasticity. Intriguingly, efficient beta cell regeneration has been observed in children with type-1 diabetes (T1D) or after pancreatectomy [19], but whether the regenerative mechanism involves alpha or delta to beta cell conversion still remains unexplored.

Alpha to beta cell conversion has also been genetically triggered by overexpression of the alpha cell fate repressor factor Pax4 in glucagon cells [20] (Figure 2a). A combination of different lineage tracing experiments showed that upon Pax4-induced alpha to beta cell conversion, a pool of duct lining progenitors is mobilised, proliferate and further replenish the alpha cells which were converted to beta cells. Interestingly, this mechanism is mimicked by chemically inhibiting the Pax4 repressor Arx using gamma-aminobutyric acid (GABA) or Artemisinin treatment [21••, 22•]. The molecular characterization of the duct lining progenitors mobilised after GABA treatment could be of special interest to shed light on potential facultative progenitors in the pancreas. In addition, since GABA is a natural occurring neurotransmitter, one immediate question arising from these findings would be whether physiological/physiopathological stimuli involving GABA increase could be the biological trigger for activating the alpha to beta cell conversion program.

### Acinar plasticity

Acinar cell plasticity has been demonstrated in the context of oncogenic transformation, inflammation and experimental induced diabetes in mouse models [13, 14] (Figure 2b). After caerulin-induced pancreatitis in rodents, acinar cells undergo transdifferentiation to a progenitor-like cell with ductal characteristics able to regenerate the damaged acinar compartment [23]. This process, termed acinar-to-ductal metaplasia (ADM) has also been observed in human primary acinar cells *ex vivo* [24]. Oncogenic Kras, inflammatory cytokines or growth factors that activate epidermal growth factor receptor (EGFR) induce ADM *in vitro* [13]. Recent evidence suggests that ADM involves epigenetic silencing of genes maintaining acinar identity and activation of genes involved in de-differentiation [25].

In addition to acinar to duct conversion, acinar cells have the potential to transdifferentiate to beta, alpha and delta cells (Figure 2b). Acinar to beta cell plasticity has been achieved *in vivo* artificially by infecting acinar cells with adenovirus overexpressing the potent embryonic proendocrine factors Ngn3/Pdx1/MafA [26, 27]. However, Ngn3 overexpression in acinar cells leads to delta-like cell differentiation, while Ngn3+MafA changes their fate to alpha-like cells. These data support the role of Ngn3 as potent endocrine fate determinant factor, while MafA and Pdx1 would be required for alpha and beta cell fate commitment. Interestingly, transient administration of epidermal growth factor (EGF) and ciliary neurotrophic factor (CNTF) to adult mice with chronic hyperglycemia efficiently stimulates the conversion of terminally differentiated acinar cells to beta cells. Cytokines-induced acinar to beta cell reprogramming involves Ngn3 reactivation and is dependent on Stat3 signalling [14], though whether Pdx1 and MafA are also required in this process is not entirely clear.

### Ductal plasticity

Perhaps one of the more controversial intercellular conversions in the pancreas is the ductal to beta cell conversion (Figure 2c). This observation traces back to the early 20th century, when clusters of beta cells budding from the ducts were reported [28]. Since then, mostly based on the location of neoformed beta cells during normal growth or regeneration (close to ducts or embedded within the ductal tree), many groups have reported the potential of ductal cells to change to a beta cell fate [29, 30, 31, 32]. During embryonic development, Pdx1-expressing progenitor cells structured in duct-like complexes are the source of endocrine and exocrine cells [4]. Ngn3 expression in Pdx1-expressing progenitors is a determinant factor for beta cell commitment [4]. Interestingly, adult ductal cells seem to acquire a progenitor state in response to injury, implying that they might regain endocrine and exocrine differentiation potential [31, 32]. Single-cell RNA-seq studies have demonstrated the existence of different subpopulations of cells in the adult ductal compartment, one of which showed a progenitor-like signature [10^••^].

However, different lineage tracing strategies in mice have produced conflicting evidence of the ability of ductal cells to transdifferentiate to beta cells. Studies from Harry Heimberg's group in 2008 demonstrated that pancreatic duct ligation (PDL), an injury model that causes acinar cell death by blockage of the main pancreatic duct, was sufficient to induce new beta cells from pancreatic ductal cells [30]. Following this study, lineage tracing of ductal cells using ductal specific promoters demonstrated that ductal cells are able to transdifferentiate to beta cells after PDL. The duct-specific promoter carbonic anhydrase II (CAII) driving CreER combined with R26-LSL-BetaGal was able to irreversibly trace adult ductal cells that had the capacity to differentiate to beta cells and other lineages upon PDL [33]. In another model using Pdx1-Cre; R26-LSL-DTR to induce expression of the diptheria toxin receptor (DTR) in pancreatic cells, depletion of acinar and beta cells with diphtheria toxin resulted in surviving ductal cells regenerating beta cells [34].

Overexpression of TGFa in ductal cells [35], pancreatic ductal deletion of Fbw7 [36], and Pax4 overexpression in alpha cells [18] have also uncovered duct to beta cell plasticity. More recently, it has been shown that inflammatory cytokines are able to induce endocrine differentiation of ductal cells by activating Stat3 and Ngn3 [12]. In sharp contrast to these findings, other genetic adult ductal lineage tracing studies using ductal specific promoters such as HNF1b, Mucin1, Sox9 and Hes1 have not observed any remarkable contribution of ductal cells to beta cell regeneration [37, 38, 39, 40]. However, in all these studies the tracing efficiency was very low in the adult ductal cells, raising the possibility that the cells more prone to transdifferentiate to beta cells were not actually traced to start with. When a Sox9-CreER line with stronger labelling capacity was used in a recent study, beta cell labelling was detected after mild hyperglycemia combined with cytokine (gastrin and EGF) treatment [41^•^].

Therefore, the different experimental data suggest that at least some of the ductal cells can serve postnatally as a beta cell source, although this process may not be the main pathway for tissue regeneration after injury.

## *In vivo* tissue plasticity towards pancreatic fates

**Figure 3:**
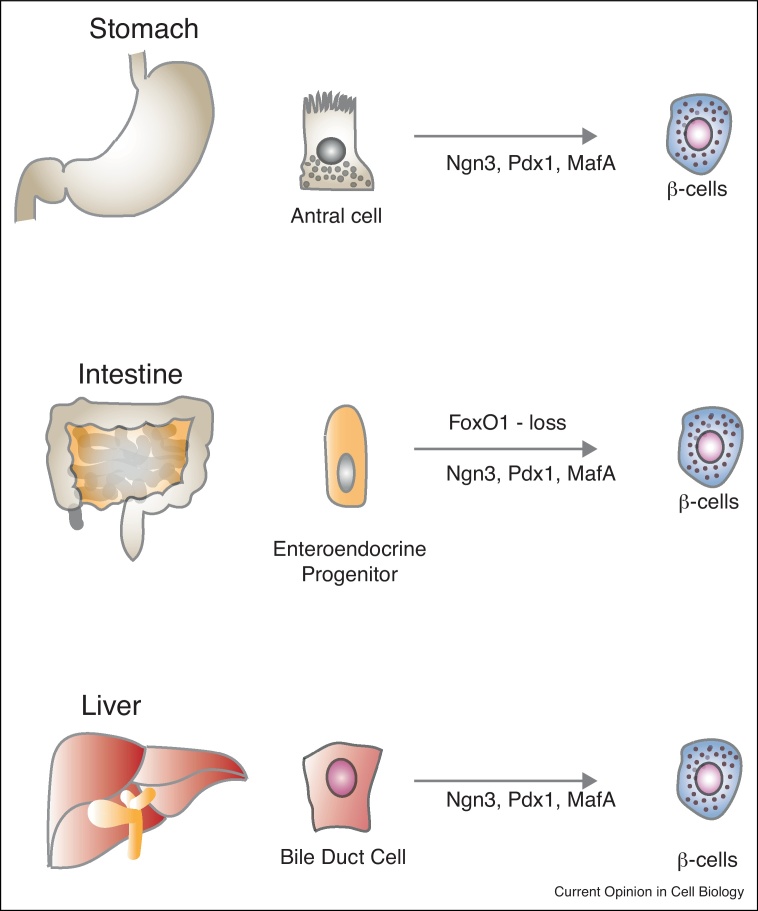
Inter-organ plasticity: intestine, stomach, liver transdifferentiation to beta-like cells. Studies have shown that stomach-residing antral cells, when genetically modified to overexpress Ngn3, Pdx1 and MafA, can give rise to beta cells which are functionally similar to native beta cells. Intestinal cells can also transdifferentiate into beta-like cells upon FoxO1 deletion or Ngn3, Pdx, MafA overexpression. Ngn3, Pdx1 and MafA ectopic expression in liver and gallbladder can activate some degree of plasticity towards pancreatic lineage.

Perhaps one of the most striking phenomena observed in rodents upon genetic modification stimuli is inter-organ plasticity (Figure 3). In particular, the gastrointestinal tract has drawn special attention in recent years. The gastrointestinal tissues (stomach and intestine) are the only endodermal-derived tissues that contain dedicated active stem/progenitor cells constantly producing differentiated cells. The pancreas, stomach and intestine are developmentally related, arising from the same embryonic domains [42]. And, similar to the pancreas, the intestine and stomach harbour scattered hormone-producing cells that originate from Ngn3-expressing progenitors [4, 43]. In contrast to the pancreas, however, Ngn3+ progenitors in the intestine and stomach are active during adulthood. Evidence has suggested that intestinal cells can transdifferentiate into beta-like cells upon FoxO1 deletion or Ngn3, Pdx1, MafA overexpression, although the functionality of such cells is incomplete [44, 45•]. However, recent studies demonstrated that antral cells in the stomach genetically modified to overexpress Ngn3, Pdx1 and MafA generate beta cells with a remarkable transcriptional similarity to bona fide pancreatic islet beta cells. This effect was restricted to antral cells in the stomach and not observed in the intestinal epithelia due to the strong expression of the intestine specific cell fate regulator cdx2 [46^••^]. Moderate plasticity towards a pancreatic cell fate after forced expression of Ngn3, Pdx1 and MafA has also been observed in other endodermal derived tissues such as the liver [47] suggesting that, even though such events probably do not occur in physiological circumstances, other organs could represent sources of regenerative beta cells derivable in the laboratory.

## *In vitro* pancreatic cell plasticity

**Figure 4:**
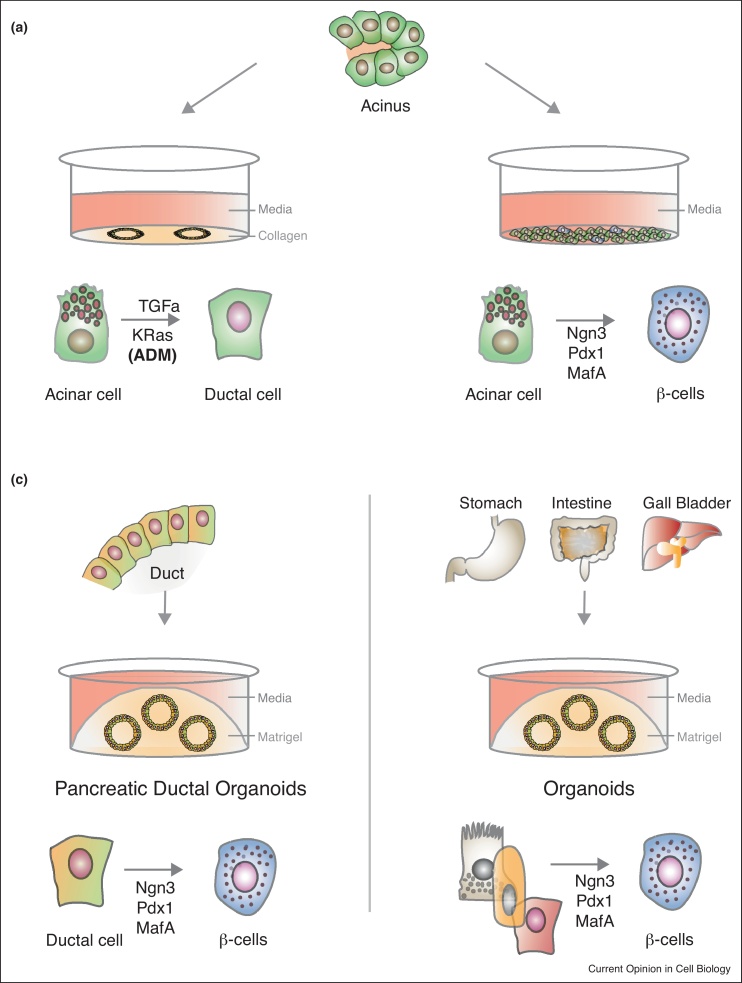
*In vitro*/*ex vivo* pancreatic cell plasticity. **(a)***In vitro* culturing of acinar cells affects their phenotype, making them susceptible to fate change. Primary acinar cells de-differentiate *ex vivo*, exhibiting a ductal-like phenotype (Sox9+, Hnf1b+ and CK19+), mimicking insult-induced ADM observed *in vivo*. Acinar cells can transdifferentiate into beta cells by either being conditioned with EGF, leukemia inhibitory factor (LIF) or ciliary neurotrophic factor (CNTF) or by inducing overexpression of Ngn3, Pdx1, MafA and Pax4. **(b)** Many adult tissue-derived primary cells can be cultured in a 3D system, forming spheres termed organoids. Pancreas, stomach, intestine and gallbladder cells can be cultured long-term as organoids, retaining their native features, and forced to transdifferentiate into beta-like cells when forced to overexpress Ngn3, Pdx1 and MafA.

The plastic potential of adult differentiated or progenitor cells is well known to be enhanced in artificial *ex vivo* culturing systems, probably due to the lack of cell fate commitment signals received *in vivo* by neighbouring cells. In particular, the ability of non-beta cells to differentiate to beta cells has been widely demonstrated (Figure 4). *In vitro* culture of human exocrine tissue or ductal cell lines with bone morphogenetic protein 7 (BMP7) or preadipocyte factor 1 (Pref-1) resulted in beta cells that were able to reverse diabetes when transplanted in diabetic mice [48, 49].

Rodent and human acinar cells are particularly prone to phenotypic change when cultured *in vitro*. Primary acinar cells rapidly lose their acinar fate to de-differentiate into a ductal-like phenotype expressing Sox9, Hnf1b and Cytokeratin 19, recapitulating the ADM observed *in vivo* upon inflammation or oncogenic stress [8, 14]. In addition, treatment with EGF, LIF or overexpression of Pdx1, MafA, Ngn3 and Pax4 enables acinar cells to differentiate to beta cells [14] (Figure 4a).

Primary cells from many adult tissues, including gut, liver, kidney, brain, stomach and many others, can be efficiently cultured in a three-dimensional matrix (organoid cultures). These organoids are proving to be an excellent tool to understand the function of tissue progenitor/stem cells. Consistent with this notion, both mouse and human pancreatic ductal cells can be cultured long-term *ex vivo* as sphere-forming organoids [50]. *Ex vivo* pancreatic organoids retain features of their originating ductal cells in the pancreas. However, different experimental conditions can uncover their dormant plasticity. Co-culture of ductal organoids with embryonic pancreatic progenitor cells is sufficient to prime ductal organoids to differentiate into beta-cells after transplantation under the kidney capsule [50]. Genetically, overexpression of Ngn3, Pdx1 and MafA is sufficient to observe beta-like cell differentiation in organoids from pancreas, intestine and gallbladder [46••, 51•] (Figure 4c). Therefore, the organoid technology could be a promising expansion platform for cells which plasticity could be explored in beta cell regeneration.

## Conclusions

Phenotypic plasticity — that is, the faculty of some cells to undergo phenotype switching to another cell type — seems to be a rather universal phenomenon, not only restricted to highly dynamic organs but also observed in poorly regenerating tissues such as the pancreas.

The concept of potential plasticity of the different pancreatic cell types in rodents has become somewhat accepted, but whether this plasticity is an essential regenerative response of the pancreas to physiological stimuli, and whether these events are shared in human pancreas is still a matter of contention.

The last decade has witnessed an expansion in the field of pancreas plasticity. It has now become apparent that all the pancreatic cell types contain different subpopulations with different plastic potential, and perhaps future lineage tracing analysis targeting different subpopulations will determine if any of those cell subtypes could act as facultative progenitors in physiological conditions.

The majority of triggers used to uncover the plasticity of different pancreatic populations are rather artificial. Most of the models used to uncover plasticity are based on physical injury or genetic manipulations and involve re-activation of embryonic factors, such as Ngn3 for the efficient conversion to beta-like cells. It still remains unexplored whether other cell fate-restricting factors are required to maintain the new fate and erase the previous fate. Artificial or not, the latest findings have opened new avenues to explore in research aiming to replenish lost beta cells in diabetic patients. Expanding and regenerating the beta cell mass and restoring the damaged cells could be an effective therapeutic approach and an important milestone in regenerative medicine that could benefit from the knowledge acquired from both artificial and physiological triggers of pancreas plasticity.

## References and recommended reading

Papers of particular interest, published within the period of review, have been highlighted as:

• of special interest
•• of outstanding interest
